# Circulating metabolites and coronary heart disease: a bidirectional Mendelian randomization

**DOI:** 10.3389/fcvm.2024.1371805

**Published:** 2024-05-21

**Authors:** Huanyu Chen, Yuxuan Huang, Guangjing Wan, Xu Zou

**Affiliations:** ^1^ The Second Clinical Medical School, Guangzhou University of Chinese Medicine, Guangzhou, China; ^2^ Department of Cardiology, Guangdong Provincial Hospital of Traditional Chinese Medicine, Guangzhou, China

**Keywords:** blood metabolites, causality, colocalization analysis, coronary heart disease, Mendelian randomization

## Abstract

**Background:**

Numerous studies have established a link between coronary heart disease and metabolic disorders. Yet, causal evidence connecting metabolites and Coronary Heart Disease (CHD) remains scarce. To address this, we performed a bidirectional Mendelian Randomization (MR) analysis investigating the causal relationship between blood metabolites and CHD.

**Methods:**

Data were extracted from published genome-wide association studies (GWASs) on metabolite levels, focusing on 1,400 metabolite summary data as exposure measures. Primary analyses utilized the GWAS catalog database GCST90199698 (60,801 cases and 123,504 controls) and the FinnGen cohort (43,518 cases and 333,759 controls). The primary method used for causality analysis was random inverse variance weighting (IVW). Supplementary analyses included MR-Egger, weighted mode, and weighted median methods. Sensitivity analyses were conducted to evaluate heterogeneity and pleiotropy. Reverse MR analysis was employed to evaluate the direct impact of metabolites on coronary heart disease. Additionally, replication and meta-analysis were performed. We further conducted the Steiger test and colocalization analysis to reflect the causality deeply.

**Results:**

This study identified eight metabolites associated with lipids, amino acids and metabolite ratios that may influence CHD risk. Findings include: 1-oleoyl-2-arachidonoyl-GPE (18:1/20:4) levels: OR = 1.08; 95% CI 1.04–1.12; *P* = 8.21E-06; 1-palmitoyl-2-arachidonoyl-GPE (16:0/20:4) levels: OR = 1.07; 95% CI 1.04–1.11; *P* = 9.01E-05; Linoleoyl-arachidonoyl-glycerol (18:2/20:4): OR = 1.08; 95% CI 1.04–1.22; *P* = 0.0001; Glycocholenate sulfate: OR = 0.93; 95% CI 0.90–0.97; *P* = 0.0002; 1-stearoyl-2-arachidonoyl-GPE (OR = 1.07; 95% CI 1.03–1.11; *P* = 0.0002); N-acetylasparagine (OR = 1.04; 95% CI 1.02–1.07; *P* = 0.0030); Octadecenedioate (C18:1-DC) (OR = 0.93; 95% CI 0.90–0.97; *P* = 0.0004); Phosphate to linoleoyl-arachidonoyl-glycerol (18:2–20:4) (1) ratio (OR = 0.92; 95% CI 0.88–0.97; *P* = 0.0005).

**Conclusion:**

The integration of genomics and metabolomics offers novel insights into the pathogenesis of CHD and holds significant importance for the screening and prevention of CHD.

## 1 Introduction

Coronary heart disease (CHD) is the predominant cardiovascular disease globally and a leading cause of mortality (1). Despite significant advances in pharmacological and surgical interventions, these therapies address only a limited number of the potential pathophysiological pathways, resulting in persistently high mortality rates in CHD patients (2–4).

In recent years, integrating metabolomics into systems biology has offered novel insights into disease mechanisms. Specifically, metabolomics plays a crucial role in elucidating the biological mechanisms underlying diseases, primarily through the identification of altered metabolites and metabolic pathways (5, 6). For instance, gut metabolites, such as trimethylamine-N-oxide (TMAO), have been shown to have a strong association with coronary heart disease (CHD). Yao et al. (7) notably discovered that elevated levels of TMAO correlate with increased incidences of major adverse cardiac events (MACE) in patients with CHD. Furthermore, a considerable number of studies have explored the potential relationship between metabolites and CHD, suggesting that specific metabolites may play roles in the onset and progression of CHD. For instance, eicosapentaenoic acid (EPA) is a crucial anti-inflammatory/anti-aggregation fatty acid, while arachidonic acid (AA) is a precursor for various pro-inflammatory/pro-aggregation mediators. Epidemiological evidence suggests that a low EPA: AA ratio is linked to heightened CHD risk (8, 9), and clinical studies demonstrate that increasing this ratio can effectively improve CHD prognosis (10). Ganna et al. identified four lipid-related metabolites with evidence for clinical utility, as well as a causal role in CHD development.^1^ Hosseinkhani et al. (11) observed that levels of circulating amino acids and acylcarnitines were partially correlated with CHD severity in postmenopausal women. Würtz et al. substantiates the value of high-throughput metabolomics for biomarker discovery and improved risk assessment.^2^ Unfortunately, existing studies examining the causal relationship between metabolites and CHD have limitations, particularly in terms of their evaluation systems needing to be more comprehensive. Nevertheless, there is still a need for more comprehensive and systematic studies to evaluate this causal relationship between blood metabolites and CHD. Given the inherent limitations of traditional observational studies, existing evidence cannot definitively establish a metabolic profile associated with the onset of CHD. While rigorous randomized controlled trials (RCTs) are the quintessence standard in evidence-based medicine for establishing causality, their implementation faces challenges due to ethical concerns, observational duration, high costs, and other logistical constraints.

Mendelian randomization (MR) studies have increasingly been utilized in investigating disease etiology. In scenarios where RCTs are not feasible, MR emerges as a highly compelling approach to ascertain causality between an exposure of interest and its corresponding outcome (12). Unlike traditional studies, MR utilizes exposure-associated single nucleotide polymorphisms (SNPs) as instrumental variables (IVs) and employs genetic proxies to assess the causal effects of exposure (13). Precisely, this IV method, as applied in Mendelian Randomization, parallels RCTs since SNPs are randomly assigned to offspring at conception (14). This significantly reduces confounding factors, as variables such as gender and age are unlikely to influence the causal effects assessed.

Given the limited understanding of the causal relationship between blood metabolites and CHD, in-depth research in this field is essential. To address this, our study collected comprehensive serum metabolome data (encompassing 1,400 types) and applied an MR analysis akin to an RCT design. This approach enabled us to comprehensively assess the causal relationship between CHD and relevant metabolites through a bidirectional MR validation. Genome-wide association studies (GWAS) can identify multiple genetic associations, but distinguishing causal variants from simple correlations remains a significant challenge. Refining these findings, the colocalization analysis offers evidence to determine whether the same causal variant may influence multiple traits. Consequently, we conducted GWAS-GWAS colocalization analysis to identify and mitigate false positive results. This was achieved by comparing gene loci across different GWAS, which ensures a more accurate identification of genuine genetic associations.

## 2 Methods and materials

### 2.1 Study design

All published GWAS received ethical approval from the appropriate institutional review boards. This study utilized only summary-level data, eliminating the need for additional ethical approval.

**Figure 1:**
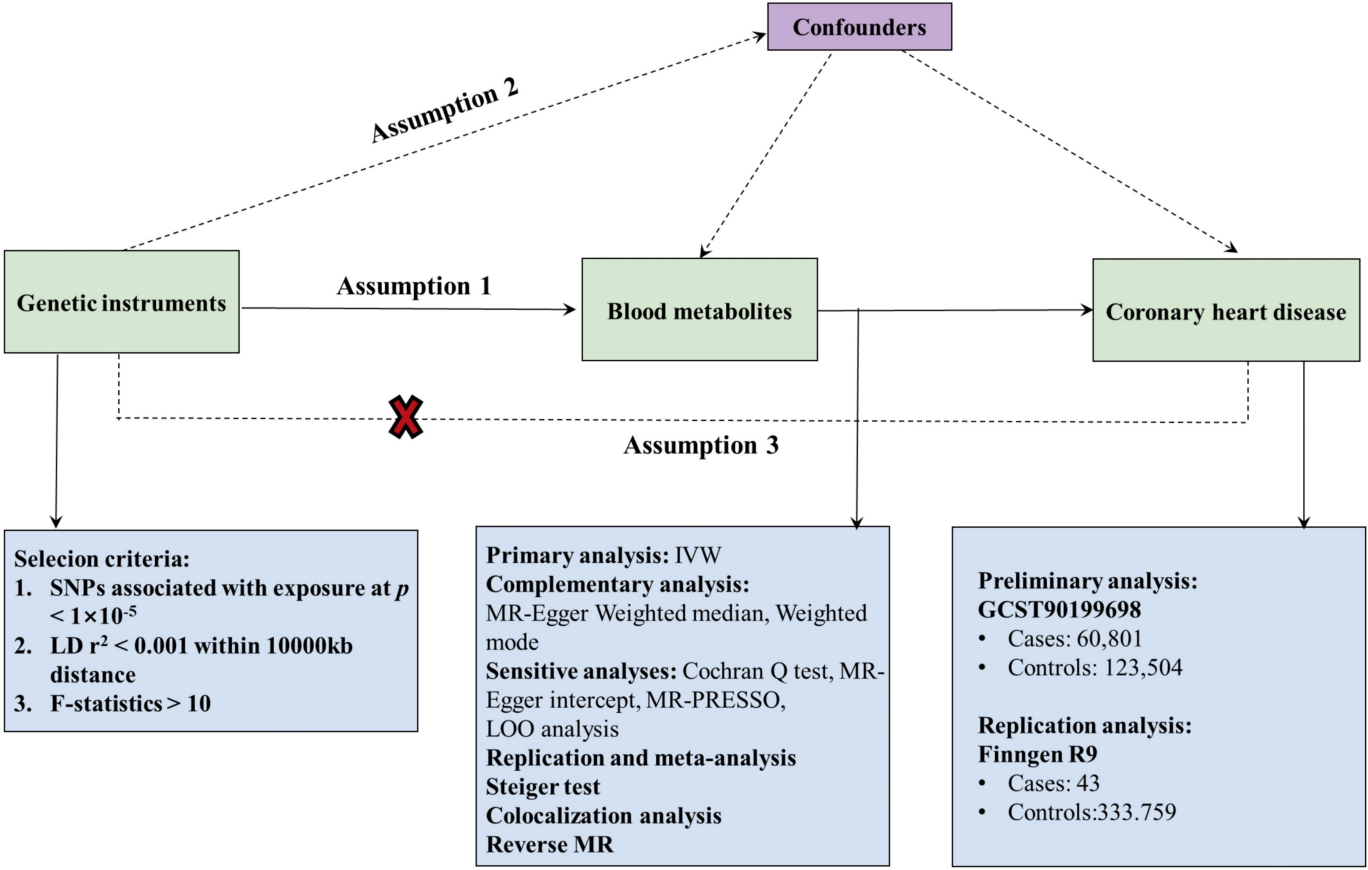
Flow chart of Mendelian randomization (MR) analysis. This overview illustrates the three fundamental hypotheses of MR analysis: Hypothesis 1 posits that the genetic instruments are strongly associated with the exposure of interest; Hypothesis 2 asserts that genetic instruments are independent of confounders; Hypothesis 3 stipulates that genetic instruments affect outcomes solely through exposure, with no direct association to outcomes. Key terms include IVW (Inverse Variance Weighting), LD (Linkage Disequilibrium), LOO analysis (Leave-One-Out Analysis), MR-PRESSO (MR-Pleiotropic Residuals Sum and Outlier), SNPs (Single Nucleotide Polymorphisms), and WM (Weighted Median).

In this study, we conducted a rigorous MR design to systematically evaluate the causal relationship between 1,400 human blood metabolites and CHD risk. A robust MR design must satisfy three key assumptions: (1) the genetic instrument exhibits a strong association with the exposure; (2) the genetic instruments are independent of confounding factors; (3) the genetic instruments influence the outcome solely through the exposure of interest (15). Due to the independence from horizontal pleiotropy inherent in the second and third hypotheses, various statistical methods can be utilized to confirm this, as detailed in relevant literature (16). In order to minimize the bias inherent in the genetic data of CHD, our study undertook both preliminary and replication analyses using two independent GWAS consortia, culminating in a meta-analysis. Figure 1 presents a schematic of this bidirectional MR study.

### 2.2 GWAS data for 1,400 serum metabolites

This research, which involves a dataset of 1,400 blood metabolites from genome-wide association studies (GWAS), is based on the study by Chen et al. (17). Genetic information for each metabolite is available at https://www.ebi.ac.uk/gwas/publications/36635386 and can be accessed through the Metabolomics GWAS Server. Specifically, the data were derived from a large-scale genome-wide association scan and high-throughput metabolic analysis performed by Chen et al. (17). Specifically, the analysis encompassed 1,091 metabolites and 309 metabolite ratios in a sample of 8,299 individuals from the Canadian Longitudinal Study of Aging (CLSA) cohort. Using genome-wide association studies of 1,091 blood metabolites and 309 metabolite ratios, researchers identified associations with 690 metabolites at 248 loci. And the association with 143 metabolite ratios at 69 sites. In the study, out of the 1,091 metabolites examined, 850 were classified into eight metabolic pathways: lipids, amino acids, cofactors and vitamins, xenobiotics, carbohydrates, nucleotides, peptides, and energy metabolism. The remaining 241 substances were identified as unknown molecules.

### 2.3 GWAS data for CHD

GWAS statistics for coronary artery disease in the primary analysis were obtained from the CARDIoGRAMplusC4D consortium. This consortium, which focuses on the replication and meta-analysis of Coronary Artery Disease (CARDIoGRAM) and Coronary Artery Disease Genetics (C4D), included 60,801 CHD cases and 123,504 control subjects (18). The consortium's data is accessible at https://gwas.mrcieu.ac.uk (19). Chronic stable angina, acute coronary syndrome, coronary artery stenosis exceeding 50%, and myocardial infarction were included in the case definition of coronary heart disease (18). To substantiate our results through replicate analysis and meta-analysis, we utilized data from the FinnGen R9 study of coronary heart disease, comprising 43,518 cases and 333,759 controls.

### 2.4 IVs selection

The selection of instrumental variables (IVs) in this Mendelian Randomization (MR) analysis adhered to three fundamental assumptions. Firstly, single nucleotide polymorphisms (SNPs) strongly associated with *P*-values less than 1 × 10^−5^ were identified for each metabolite, establishing the genome-wide significance threshold. Secondly, we employed the clustering procedure in R software to discern independent variants, defining linkage disequilibrium (LD) as an R^2^ of less than 0.001 within a 500-kilobase (kb) distance. This criterion has been extensively adopted in prior research (20, 21). Finally, to verify the suitability of the selected SNPs as instrumental variables, we calculated their proportion and *F*-statistic for each metabolite. Generally, an *F*-statistic exceeding ten was considered the threshold for selecting robust instrumental variables in subsequent analyses (22).

### 2.5 MR analysis

This study investigated the causal relationship between metabolites and CHD using bidirectional MR analysis with the standard IVW method. Although propensity score matching (PSM), difference-in-differences (DiD), and regression discontinuity design (RDD) are analytical methods used in epidemiology and economics to infer causality, PSM is limited to accounting for observed and measured confounding factors. Any unmeasured confounders could still bias the results; moreover, the quality of the match significantly influences the estimates, and a perfect match in propensity scores does not ensure the balance of all critical covariates. DiD assumes parallel trends, meaning that, in the absence of treatment, the treatment and control groups' average outcomes would follow a parallel trajectory over time. Any deviation from this assumption could lead to biased estimates. RDD's applicability is confined to individuals near the cutoff point, limiting result generalization. Furthermore, if individuals can manipulate their position relative to the cutoff, the design's integrity may be compromised.

The selection of these methods is influenced by various factors, such as the data's nature, the research question, the validity of underlying assumptions, and the method's applicability in specific contexts. In contrast, MR is particularly suited for assessing the causal effects of biomarkers or exposures that are challenging to manipulate directly. This is because MR employs genetic variation as an instrumental variable. Given that genetic variation inherently exists, MR is effective in determining the causal effects of lifetime exposures, thereby validating the methodological choice of MR in this study.

While IVW assumes the validity of all genetic variants, making it highly effective for MR estimation, it is not without challenges, primarily its susceptibility to pleiotropy. Thus, IVW served as the primary method for identifying CHD-related metabolites, complemented by various assessment methods to address its limitations. Secondary evaluation methods, including the weighted model, MR Egger, and weighted median (WM), were employed further to scrutinize significant metabolites (IVW *P* < 0.05), enhancing the robustness of the MR results.

Sensitivity analyses were conducted to evaluate the impact of MR assumptions on the significance estimates and to mitigate potential biases from horizontal pleiotropy and heterogeneity. These analyses included: (1) heterogeneity assessment using *Q*-tests with IVW and MR Egger methods; (2) estimation of horizontal pleiotropy via the MR Egger intercept; (3) application of additional methods like the weighted median and modulus for hypothesis testing reliability; (4) single SNP analysis and a retention test for assessing associations (23, 24). Additionally, heterogeneous SNPs were re-examined using MR-PRESSO. For result robustness, a leave-one-out (LOO) analysis was conducted, sequentially excluding each SNP, to determine if the results were disproportionately influenced by individual SNPs.

Therefore, we identified potential candidate metabolites associated with CHD development based on the following items: (1) the *P*-value of the primary analysis was significant, with the IVW method yielding a *P*-value below 0.05; (2) the directions and magnitudes of the results were consistent across the four MRI methods; (3) the MR results exhibited neither heterogeneity nor horizontal pleiotropy; (4) no single SNP significantly influenced the Mendelian Randomization estimates.

### 2.6 Replication and meta-analysis

To verify the robustness of the candidate metabolites, we repeated the IVW analysis in an additional CHD cohort. There are publicly accessible databases resulting from genome-wide association studies, one of which is the FinnGen research project, which has provided genetic insights from phenotypically good isolated populations (25). The replication analysis for CHD utilized GWAS data from the latest R9 publication (43,518 cases and 333,759 controls) provided by the FinnGen Consortium, available at https://r9.finngen.fi/. Through a meta-analysis of two MR Analyses, we finally identified blood metabolites associated with a causal link to CHD.

### 2.7 Colocalization analysis

We conducted a colocalization analysis with the coloc R package to further explore if the relationship between the identified metabolites and CHD was due to a shared causal variant (26). This involved examining regional loci within 1,000 KB above and below the lead SNP in the exposure data, thereby mitigating the risk of reinforcing spurious associations between the two phenotypes. In the Bayesian framework, the coloc approach evaluated posterior probabilities for five hypotheses (H0, H1, H2, H3, H4) at each variant locus: (1) no association with either trait; (2) association with only trait 1; (3) association with only trait 2; (4) associations with both traits due to different causal variants; and (5) both traits sharing a common causal variant (27). We employed default priors (p1 = 1 × 10^–4^, p2 = 1 × 10^–4^, p12 = 1 × 10^–5^) for the colocalization analysis. The presence of a posterior probability exceeding 80% for H4 (PP4) under diverse prior and window conditions was deemed compelling evidence of colocalization.

## 3 Results

### 3.1 Analyzing the impact of 1,400 blood metabolites on coronary heart disease

**Figure 2:**
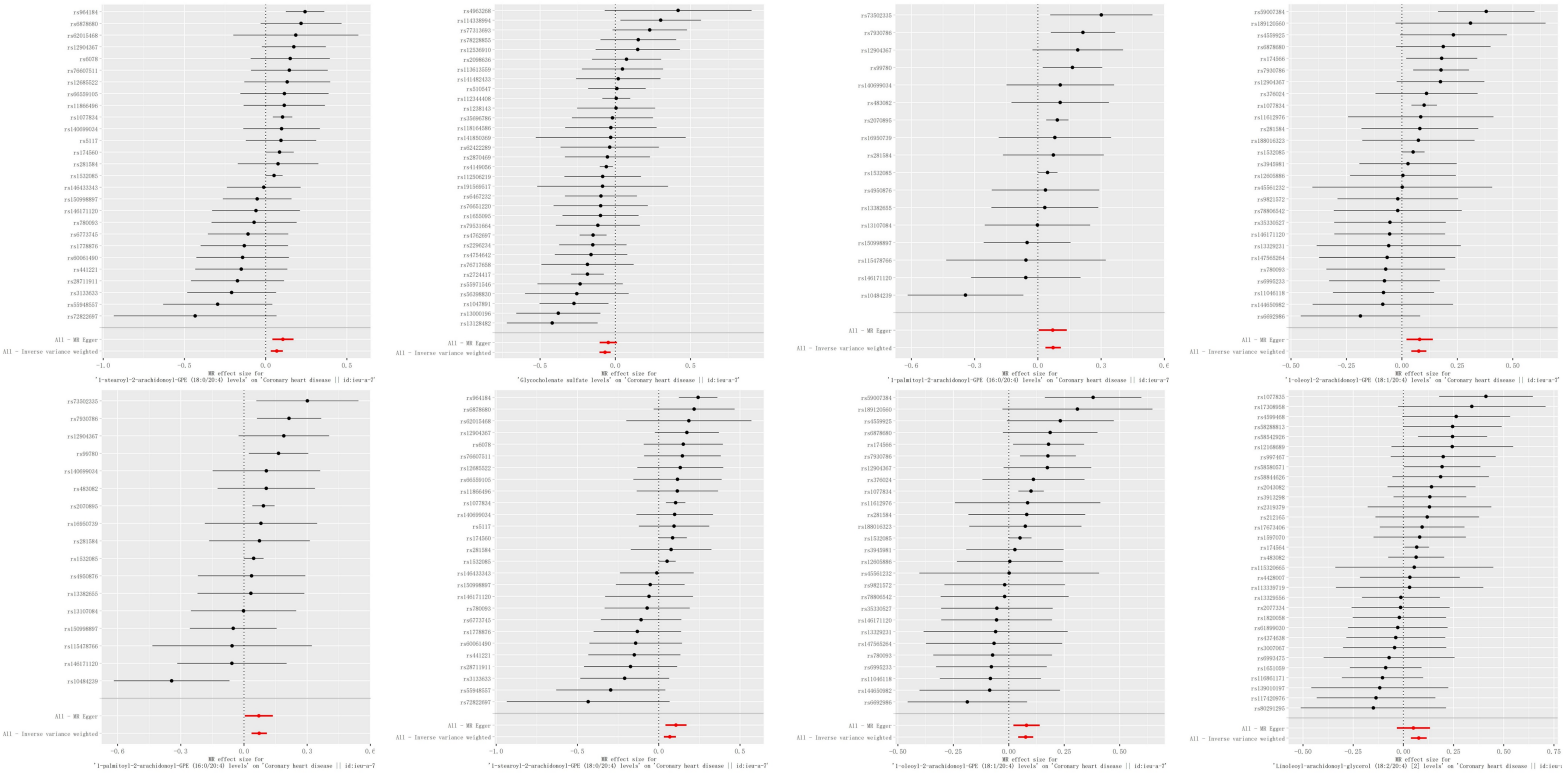
Forest plots of causal relationships. Forest plots illustrating the causal relationships between blood metabolites and coronary heart disease (CHD), derived from inverse variance weighted (IVW) analysis. Key terms: CI (confidence interval), IVW (inverse variance weighting), OR (odds ratio), and SNPs (single nucleotide polymorphisms).

**Figure 3:**
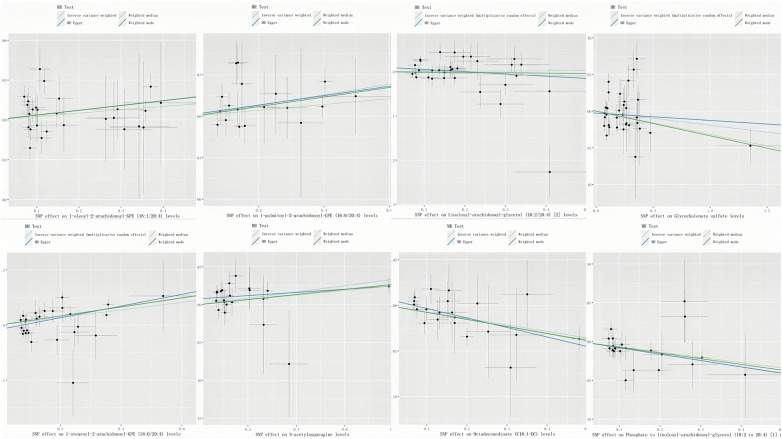
Scatter plots of significant associations. Scatter plots depicting significant associations (IVW-derived *P* < 0.05) with consistent directionality. Key term: SNP (single nucleotide polymorphism).

A genome-wide significance threshold of *P* < 1 × 10^−5^ was employed to identify strongly associated SNPs in conjunction with screening 1,400 serum metabolites. Within the filtered IVs, a total of 34,931 SNPs were identified. All SNPs associated with metabolites demonstrated *F*-statistics greater than 10, suggesting the exclusion of weak analytical tools, as detailed in Supplementary Table S2. The IVW analysis identified 113 metabolites potentially causing effects on CHD (*P* < 0.05 for IVW), comprising 60 protective and 53 risk factors for CHD. Subsequent IVW analysis identified 113 metabolites potentially impacting Coronary Heart Disease (CHD) (*P* < 0.05 for IVW), comprising 60 protective factors and 53 risk factors. The most notable metabolite was 1-oleoyl-2-arachidonoyl-GPE (18:1/20:4) (*P* = 8.21E-06), followed by 1-palmitoyl-2-arachidonoyl-GPE (16:0/20:4) (*P* = 9.01E-05) and linoleoyl-arachidonoyl-glycerol (18:2/20:4) (*P* = 1.12E-04), as detailed in Supplementary Table S7. Of the 113 metabolites analyzed, 42 remained unnamed. Supplementary Table S8 presents the chemical classifications of 102 known metabolites, encompassing amino acids, carbohydrates, dipeptides, lipids, nucleotides, metabolite ratios, and xenobiotics. From these, eight metabolites (FDR < 0.1)meeting stringent screening criteria were identified as prime candidates through complementary and sensitivity analyses (refer to Table 1 and Supplementary Table S3), including 1-oleoyl-2-arachidonoyl-GPE (18:1/20:4) levels: OR = 1.08; 95% CI 1.04–1.12; *P* = 8.21E-06; 1-palmitoyl-2-arachidonoyl-GPE (16:0/20:4) levels: OR = 1.07; 95% CI 1.04–1.11; *P* = 9.01E-05; Linoleoyl-arachidonoyl-glycerol (18:2/20:4): OR = 1.08; 95% CI 1.04–1.22; *P* = 0.0001; Glycocholenate sulfate: OR = 0.93; 95% CI 0.90–0.97; *P* = 0.0002; 1-stearoyl-2-arachidonoyl-GPE (OR = 1.07; 95% CI 1.03–1.11; *P* = 0.0002); N-acetylasparagine (OR = 1.04; 95% CI 1.02–1.07; *P* = 0.0030); Octadecenedioate (C18:1-DC)(OR = 0.93; 95% CI 0.90–0.97; *P* = 0.0004); Phosphate to linoleoyl-arachidonoyl-glycerol (18:2–20:4) (1) ratio (OR = 0.92; 95% CI 0.88–0.97; *P* = 0.0005) (Figures 2, 3). The IVW estimates showed significance (*P* < 0.05) and exhibited consistent direction and magnitude across IVW, Weighted median, Weighted mode, and MR-Egger analyses. The MR-PRESSO results indicated no heterogeneity in SNPs after outlier removal (Supplementary Table S6), and both the Cochran *Q* test and the MR-Egger intercept test supported the absence of heterogeneity and pleiotropy (*P* < 0.05) (Table 1). The Leave-One-Out (LOO) analysis confirmed that individual SNPs did not bias MR estimates (Supplementary Figures S1–S8). All estimates demonstrated a power exceeding 0.8. These eight serum metabolites warrant further investigation. Furthermore, we conducted a Steiger test to address potential reverse causality bias (Supplementary Table S5) (28). In cases of coronary heart disease, a scenario where the interpretable variance explained by IVs exceeds that of the blood metabolites could result in an incorrect inference of causal direction.

**Table 1:** Complementary and sensitivity analyses of blood metabolites for causal association with CHD.

Metabolites	Method	pval	or	or_lci95	or_uci95	Heterogeneity			Pleiotropy	
						Q	Q_df	Q_pval	pval	egger_intercept
1-oleoyl-2-arachidonoyl-GPE (18:1/20:4) levels	Inverse variance weighted	8.21E-06	1.079060344	1.043571681	1.115755867	33.13412716	26	0.158242141	0.879651954	−0.000770434
	MR Egger	0.01370038	1.083125487	1.021049566	1.148975388					
	Weighted median	0.001674914	1.073624152	1.027092847	1.122263507					
	Weighted mode	0.001139778	1.076739152	1.034890661	1.120279895					
1-palmitoyl-2-arachidonoyl-GPE (16:0/20:4) levels	Inverse variance weighted	9.01E-05	1.074834452	1.036702573	1.114368894	23.61323878	16	0.098298694	0.951883524	0.000456903
	MR Egger	0.054342205	1.07299739	1.004304597	1.146388658					
	Weighted median	0.00140512	1.07349369	1.027772478	1.121248844					
	Weighted mode	0.002254746	1.069041218	1.03118466	1.108287556					
Linoleoyl-arachidonoyl-glycerol (18:2/20:4) (2) levels	Inverse variance weighted	0.000112339	1.079652367	1.038468231	1.122469806	39.06761928	31	0.151443749	0.472935093	0.004707366
	MR Egger	0.244041965	1.051102597	0.968150776	1.141161786					
	Wighted mediane	0.021663415	1.06835386	1.00972833	1.13038323					
	Weighted mode	0.040745461	1.067045224	1.005341746	1.132535791					
Glycocholenate sulfate levels	Inverse variance weighted (multiplicative random effects)	0.000239145	0.933817987	0.90031836	0.96856409	53.52873664	32	0.009898288	0.343510002	−0.005343631
	MR Egger	0.118812943	0.954223516	0.901128333	1.01044711					
	Weighted median	0.003261471	0.93935781	0.901011751	0.979335835					
	Weighted mode	0.006016271	0.941394102	0.904272183	0.980039938					
1-stearoyl-2-arachidonoyl-GPE (18:0/20:4) levels	Inverse variance weighted (multiplicative random effects)	0.000250978	1.070702112	1.032252615	1.110583781	41.92502193	26	0.024989042	0.178739379	−0.007994011
	MR Egger	0.003459171	1.111777142	1.042525293	1.185629186					
	Weighted median	0.00014622	1.08618925	1.040814067	1.133542604					
	Weighted mode	0.000231226	1.085349871	1.045293498	1.126941231					
N-acetylasparagine levels	Inverse variance weighted	0.000346176	1.04254463	1.019019906	1.066612438	17.39354019	21	0.686992182	0.058943149	0.007652884
	MR Egger	0.191850479	1.021233041	0.990568719	1.052846616					
	Weighted median	0.017149774	1.031452968	1.005517433	1.058057464					
	Weighted mode	0.013111041	1.033971256	1.009289775	1.059256307					
Octadecenedioate (C18:1-DC) levels	Inverse variance weighted	0.000352369	0.931052693	0.895275605	0.968259508	14.66680392	20	0.795145137	0.206904764	0.007061894
	MR Egger	0.004274841	0.900862631	0.845785382	0.95952649					
	Weighted median	0.003838789	0.926731617	0.880138019	0.975791832					
	Weighted mode	0.013718589	0.925459942	0.874890289	0.978952577					
Phosphate to linoleoyl-arachidonoyl-glycerol (18:2–20:4) (1) ratio	Inverse variance weighted	0.000499475	0.922257244	0.881174701	0.965255156	24.72741528	21	0.259162896	0.970850495	0.000243863
	MR Egger	0.099554274	0.920846336	0.838583093	1.011179431					
	Weighted median	0.026350248	0.934255615	0.879837094	0.992039959					
	Weighted mode	0.032084906	0.928958185	0.872314569	0.989279946					

### 3.2 Impact of coronary artery disease on 1,400 blood metabolites (reverse MR)

Given the absence of heterogeneity and the limitations of the measurement instrument, we employed the IVW method as our primary estimation approach. In conducting a reverse Mendelian Randomization (MR) Analysis to explore the causal effect of CHD on circulating metabolites, we identified 50 metabolites significantly associated with CHD (*P* < 0.05). These included various lipids, nucleotides, carbohydrates, and amino acids, as detailed in Supplementary Table S11. Notably, the ratio of Adenosine 5′-diphosphate (ADP) to N-palmitoyl-sphingosine (d18:1–16:0) showed the most significant association (*P* = 0.00033809), followed by Adenosine 5′-diphosphate alone (*P* = 0.003389943). Seven of these 50 metabolites identified from the reverse analysis were unnamed. Unfortunately, none of the metabolites passed the false discovery rate (FDR) test, as reported in Supplementary Table S11.

### 3.3 Replication and meta-analysis

**Figure 4:**
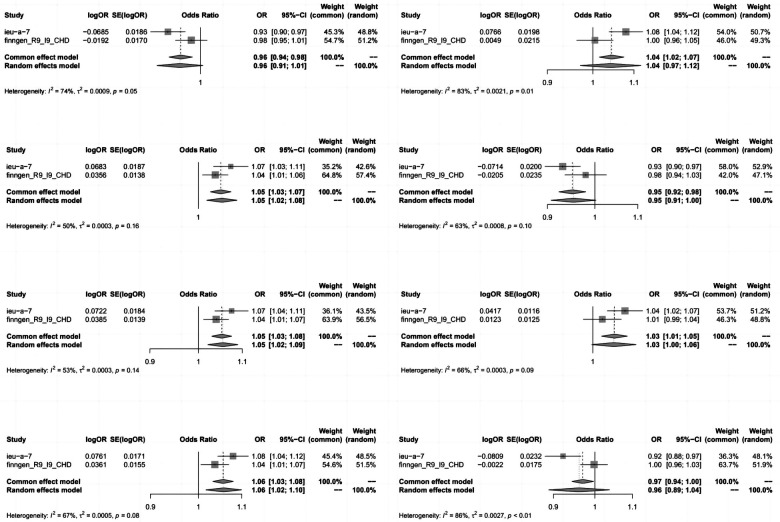
Meta-analysis of the causal relationships between metabolites and coronary heart disease. Key terms include 95% CI (95% confidence interval) and OR (odds ratio). In the heterogeneity column, the *P*-values and *I*^2^ indicate the heterogeneity test results. A *P*-value >0.1 suggests no significant heterogeneity, while <0.1 indicates notable heterogeneity. Lower *I*^2^ values denote higher reliability in the combined effect results. Typically, *I*^2^ ranges of 0%–25%, 25%–50%, 50%–75%, and 75%–100% correspond to no, mild, moderate, and severe heterogeneity, respectively.

To enhance the robustness of our estimates, we replicated the MR analysis using GWAS data from another study on CHD. In this additional GWAS dataset for CHD, we consistently observed a similar pattern in the candidate metabolites. The meta-analysis further identified eight serum metabolites significantly associated with coronary artery disease (Figure 4). These included high levels of 1-oleoyl-2-arachidonoyl-GPE (18:1/20:4) (OR 1.06, 95% CI 1.03–1.08), 1-palmitoyl-2-arachidonoyl-GPE (16:0/20:4) (OR 1.05, 95% CI 1.03–1.08), linoleoyl-arachidonoyl-glycerol (18:2/20:4) (2) (OR 1.04, 95% CI 1.02–1.07), 1-stearoyl-2-arachidonoyl-GPE (18:0/20:4) (OR 1.05, 95% CI 1.03–1.07), N-acetylasparagine (OR 1.03, 95% CI 1.01–1.05), and octadecenedioate (C18:1-DC) (OR 0.73, 95% CI 0.58–0.92, *P* = 0.008) which increased susceptibility to CHD. Conversely, high levels of octadecenedioate (C18:1-DC) (OR 0.95, 95% CI 0.92–0.98) were found. Utilizing the FinnGen coronary artery disease database, glycocholenate sulfate (Heterogeneity: *I*^2^ = 74%, *τ*^2^ = 0.0009, *P* = 0.05) and the 2-phosphate to linoleoyl-arachidonoyl-glycerol (18:2–20:4) (1) ratio (Heterogeneity: *I*^2^ = 86%, *τ*^2^ = 0.0027, *P* < 0.01) demonstrated significant heterogeneity and inconsistent directions.

### 3.4 Colocalization analysis

For the eight known metabolites identified as being associated with coronary heart disease risk in this study, we conducted a colocalization analysis to assess their relationship with CHD outcomes. This analysis utilized exposure data from within a 1,000-kb range of the lead SNP to ensure that the observed associations were not confounded by the same causal variant within a region. This approach aimed to minimize the likelihood of a spurious association between the two phenotypes (refer to Supplementary Table S10). The colocalization analysis results indicated that the associations between CHD and the eight known metabolites were unrelated to shared causal variant sites. Supplementary Figures S9–S16 depict the regional associations observed in the colocalization results.

## 4 Discussion

By integrating two large-scale GWAS datasets, we employed a rigorous two-sample MR design to explore the bidirectional causality between 1,400 blood metabolites and genetic proxies for CHD. Our analysis identified genetically determined high levels of 1-oleoyl-2-arachidonoyl-GPE (18:1/20:4), 1-palmitoyl-2-arachidonoyl-GPE (16:0/20:4), linoleoyl-arachidonoyl-glycerol (18:2/20:4), 1-stearoyl-2-arachidonoyl-GPE (18:0/20:4), N-acetylasparagine, and octadecenedioate (C18:1-DC) as risk factors for CHD. Conversely, high levels of octadecenedioate (C18:1-DC), glycocholenate sulfate, and the phosphate to linoleoyl-arachidonoyl-glycerol (18:2/20:4) ratio were identified as protective factors against CHD. Our study did not identify any blood metabolites with bidirectional effects. Furthermore, GWAS-GWAS co-localization analysis provided robust evidence of the causal relationships unaffected by overlapping SNPs.

In recent years, the substantial global health burden resulting from the high morbidity and mortality associated with CHD has emphasized the pressing need for early screening and preventive strategies (29, 30). The advent of metabolomics technology has heightened interest in exploring the role of metabolites in CHD. Previous clinical studies have indicated specific changes in serum metabolites among CHD patients (31–33). For instance, Fan et al. (3) reported downregulation of lysophosphatidylcholine, lysophosphatidylethanolamine 18:2, and phosphatidylethanolamine in patients with nonobstructive coronary atherosclerosis (NOCA) compared to those with normal coronary arteries, and an upregulation of sphingosine. Sphingosine expression in plants is up-regulated (33). While previous studies have convincingly demonstrated the involvement of metabolites in the biological mechanisms of CHD and their therapeutic potential, their contribution to the early screening and prevention of CHD remains constrained by unclear causal relationships. Therefore, we conducted a pivotal MR study to clarify the causal links between blood metabolites and CHD, including the associated metabolic pathways, aiming to guide CHD screening and treatment strategies.

This MR study identified three blood metabolites—octadecenedioate (C18:1-DC), glycocholenate sulfate, and the phosphate to linoleoyl-arachidonoyl-glycerol (18:2–20:4) ratio—as potential reducers of CHD risk. Unfortunately, there is limited research on octadecenedioate (C18:1-DC) and its effects related to CHD, warranting further investigation into this association. Glycocholenate sulfate, a specific bile acid metabolite, has limited direct studies examining its relationship with CHD. However, disorders in bile acid metabolism can disrupt cholesterol homeostasis, a critical factor in the development of atherosclerosis—an important CHD risk factor (34). Regarding glycocholenate sulfate, Alonso et al. (35, 36) reported that it may increase atrial fibrillation (AF) risk in a community cohort with atherosclerosis risk. The association between the phosphate to linoleoyl-arachidonoyl-glycerol (18:2/20:4) ratio and coronary heart disease has not been previously reported in the literature. However, clinical studies indicate a significant association between phosphate levels and the risk of cardiovascular events in patients with coronary heart disease undergoing percutaneous coronary intervention (PCI) (37). Linoleoyl-arachidonoyl-glycerol (18:2/20:4) (2), a diacylglycerol (DAG) comprising linoleic and arachidonic acids, plays a role in lipid metabolism and signaling pathways. DAG pathways are believed to influence metabolic disorder risk and play a key role in lipid-induced insulin resistance. Nevertheless, the precise biological mechanism of DAG in CHD has yet to be fully elucidated (38).

We also confirmed that higher levels of 1-oleoyl-2-arachidonoyl-GPE (18:1/20:4), 1-palmitoyl-2-arachidonoyl-GPE (16:0/20:4), linoleoyl-arachidonoyl-glycerol (18:2/20:4) (2), 1-stearoyl-2-arachidonoyl-GPE (18:0/20:4), N-acetylasparagine, and octadecenedioate (C18:1-DC) are detrimental to CHD. To date, there have been no reports linking 1-oleoyl-2-arachidonoyl-GPE (18:1/20:4), 1-palmitoyl-2-arachidonoyl-GPE (16:0/20:4), and 1-stearoyl-2-arachidonoyl-GPE (18:0/20:4) with CHD. Only one MR study has demonstrated a positive association between 1-stearoyl-2-arachidonoyl-GPE (18:0/20:4) and smoking, along with its mediation of the BMI-increasing effect (39). It is well-known that smoking and increased BMI are significant risk factors for coronary heart disease. 1-oleoyl-2-arachidoniyl-GPE (18:1/20:4), 1-palmitoyl-2-arachidoniyl-GPE (16:0/20:4), linoleoyl-arachidoniyl-glycerol (18:2/20:4) (2), and 1-stearoyl-2-arachidoniyl-GPE (18:0/20:4) are specialized forms of phosphatidylethanolamine (PE), containing arachidonic acid, which participate in various biological functions, including cell membrane structure and signal transduction, and are associated with cardiovascular diseases (40). Arachidonic acid, a type of *ω*-6 polyunsaturated fatty acid, has a controversial role in coronary heart disease. High levels can promote inflammation, a critical factor in cardiovascular diseases like atherosclerosis and thrombosis (41). Therefore, the specific impact of phosphatidylethanolamine (PE) on heart health, as mentioned, will relate to its effects on membrane dynamics and cellular signaling pathways. N-acetylasparagine, an amino acid derivative highly expressed in breast cancer, may enhance preoperative diagnostic efficiency by detecting specific serum metabolites (42). Compared to other amino acids and their derivatives, N-acetylasparagine has received less attention in mainstream cardiovascular research. However, N-acetylation, a common form of amino acid modification, can significantly affect protein function and metabolism. The role of N-acetylated amino acids in cardiovascular health might relate to their impact on protein activity, signaling pathways, and cytoprotective mechanisms (43). No literature reports on Octadecenedioate (C18:1-DC) have been retrieved to date. Octadecenedioate is a monounsaturated dicarboxylic acid, and the link between monounsaturated fatty acids (MUFAs) and cardiovascular health remains controversial. It is generally believed that MUFAs can improve lipid profiles, yet prospective evidence regarding the relationship between MUFA intake and CHD risk remains limited and controversial (44, 45). Dicarboxylic acid is a typical byproduct of fatty acid oxidation. Elevated levels of dicarboxylic acid may indicate altered lipid metabolism, a recognized trigger for atherosclerosis and CHD (46). Furthermore, dicarboxylic acid may play an indirect role in the pathogenesis of these conditions, with endothelial dysfunction and inflammation being vital factors in the development of atherosclerosis and CHD (47). Both monounsaturated fatty acids (MUFAs) and dicarboxylic acids are related to these pathogenic processes. In summary, while these metabolites are closely associated with CHD, their specific effects warrant detailed exploration under experimental conditions.

This study has notable strengths. Primarily, it stands as one of the most extensive and systematic investigations into the causal association between CHD and circulating metabolites, analyzing 1,400 blood metabolites. Additionally, a thorough MR analysis was conducted to mitigate the limitations seen in prior research, such as reverse causation and confounding factors. In particular, diverse methods were employed to derive persuasive estimates that address potential violations of MR assumptions. The consistency and sensitivity analyses conducted across four MR studies regarding the directionality affirm the stability of our findings. Moreover, the inclusion of supplementary GWAS data in the replication and meta-analyses provides additional evidence supporting the robustness of our conclusions. While the outcomes of the replication analysis are not exclusively ascribable to differences in sample size, they reveal a directionality aligning with the primary analysis, thus reducing the likelihood of chance findings. Co-localization analysis was also undertaken, reinforcing the credibility of our results. Furthermore, bidirectional MR analysis was utilized to more thoroughly explore the causal link between metabolites and CHD, as well as the roles of metabolic anomalies in the pathogenesis and progression of CHD.

The primary limitation of this study is the limited number of SNPs applied in the whole-genome exposure assessment. To overcome this constraint, we applied a slightly relaxed threshold for MR analysis, a common practice in analogous studies. Importantly, all selected SNPs exhibited *F*-statistics exceeding 10, indicating the robustness of our IVs. It is worth highlighting that the Steiger test results, which support a consistent causal direction, enhance the credibility of our approach with the relaxed threshold. Secondly, to address racial disparities, our MR analysis predominantly utilized GWAS data from individuals of European ancestry. This approach raises the question of whether our findings are applicable to other populations, warranting further exploration and validation. A third limitation involves the exclusion of certain key metabolites and pathways that are not identified or annotated in the pathway database. Therefore, these unexplored metabolites require in-depth investigation. Furthermore, although MR analysis offers valuable etiological insights, validating our findings through rigorous randomized controlled trials and basic research before clinical application is crucial.

## 5 Conclusion

In summary, this MR study elucidated the causal role of eight blood metabolites in CHD. These discoveries provide valuable insights into CHD's early screening, prevention, and therapeutic strategies, as well as the design of forthcoming clinical studies. Furthermore, this integrated MR analysis, which combines genomics and metabolomics, serves as a guiding framework for investigating the etiology and pathogenesis of CHD.

## Data Availability

The original contributions presented in the study are included in the article/**Supplementary Material**, further inquiries can be directed to the corresponding author.

## Footnotes

^1^Ganna A, Salihovic S, Sundström J, et al. Large-scale metabolomic profiling identifies novel biomarkers for incident coronary heart disease [J]. PLoS Genet, 2014, 10(12): e1004801.

^2^Würtz P, Havulinna AS, Soininen P, et al. Metabolite profiling and cardiovascular event risk: a prospective study of 3 population-based cohorts [J]. Circulation, 2015, 131(9): 774-85.
