# Supplementary material for: Circulating metabolites and coronary heart disease: a bidirectional Mendelian randomization
Source: Front Cardiovasc Med. 2024 May 21;11:1371805. doi: 10.3389/fcvm.2024.1371805 (PMC11148779; doi:10.3389/fcvm.2024.1371805)
Supplement: Supplementary file 2 [file Datasheet1.docx]

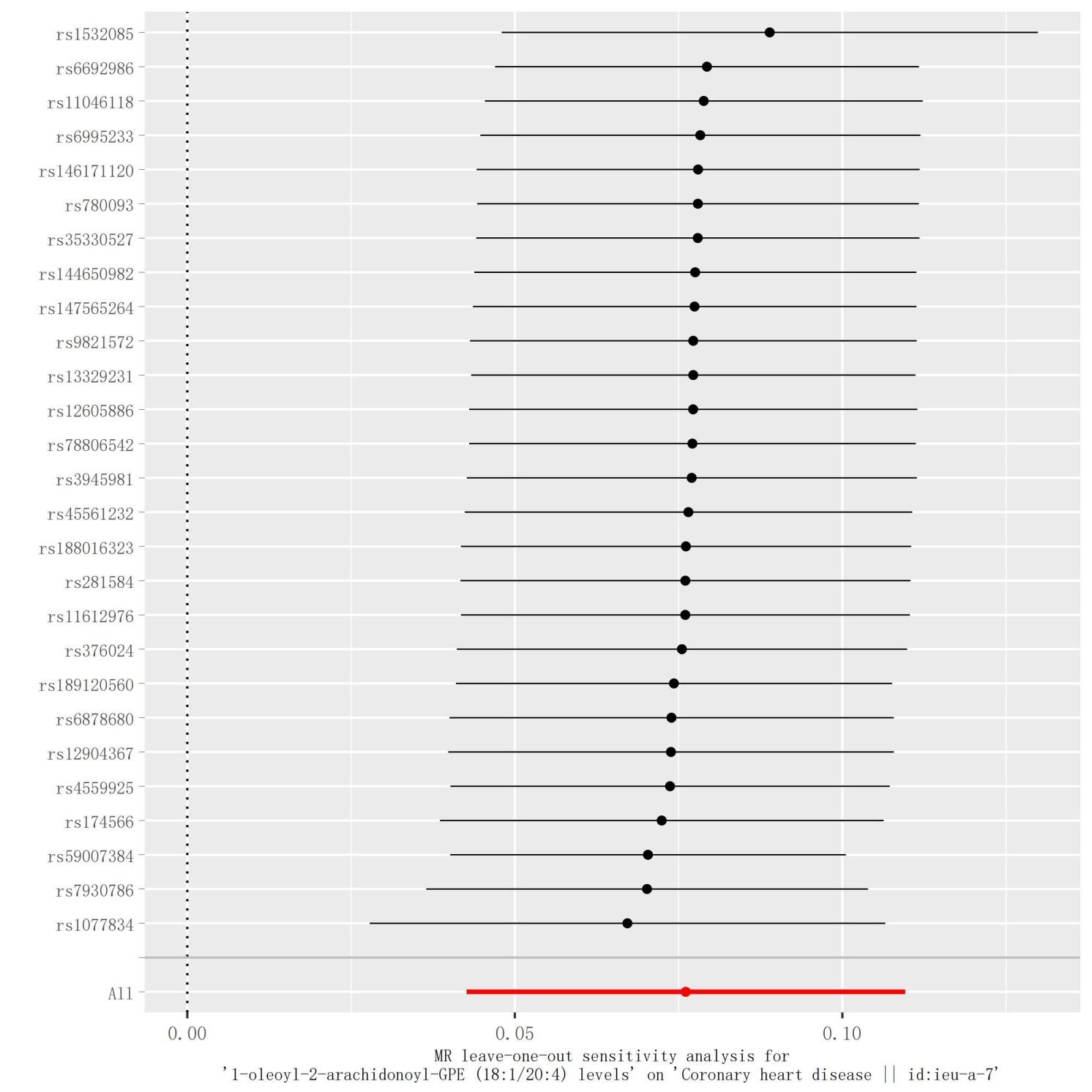


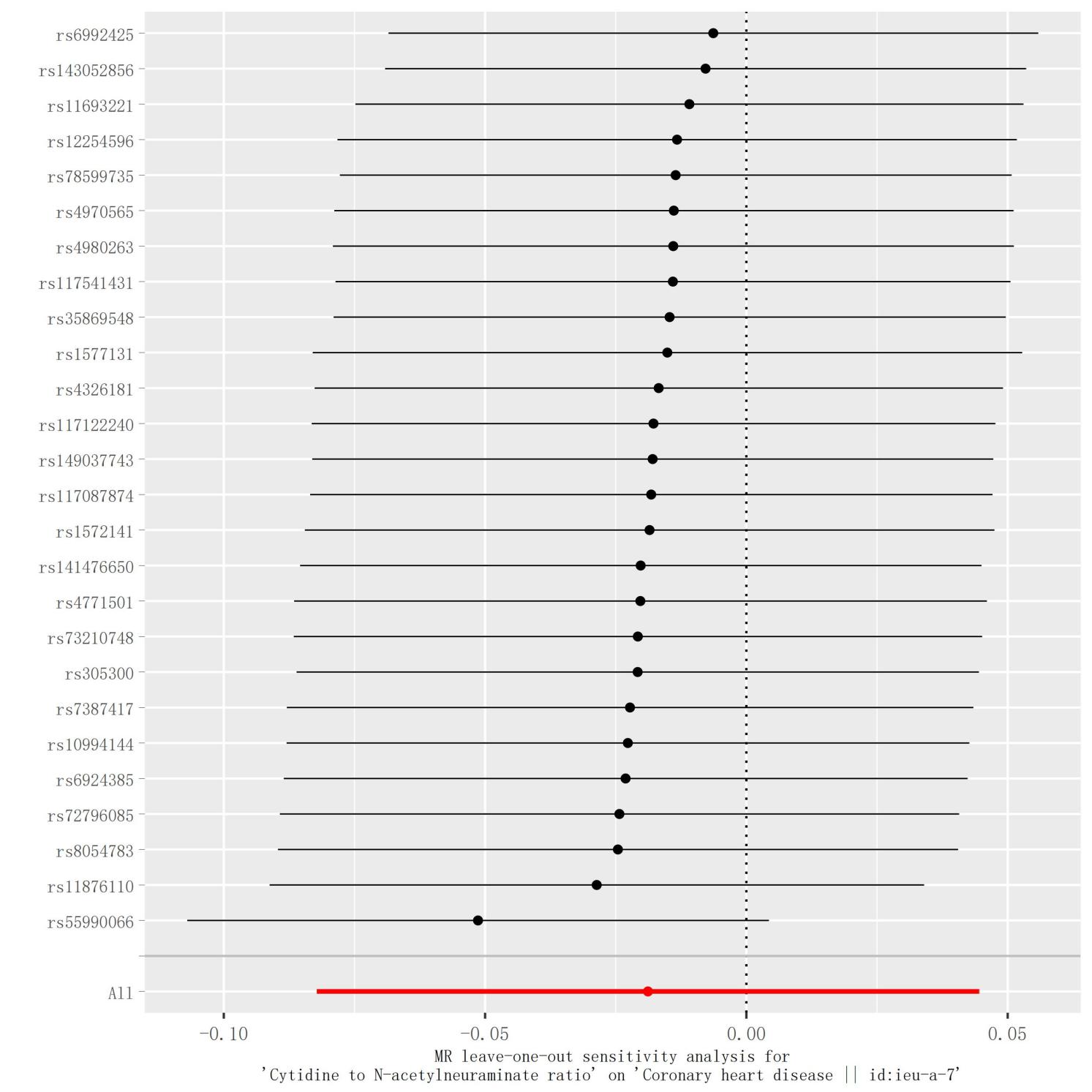

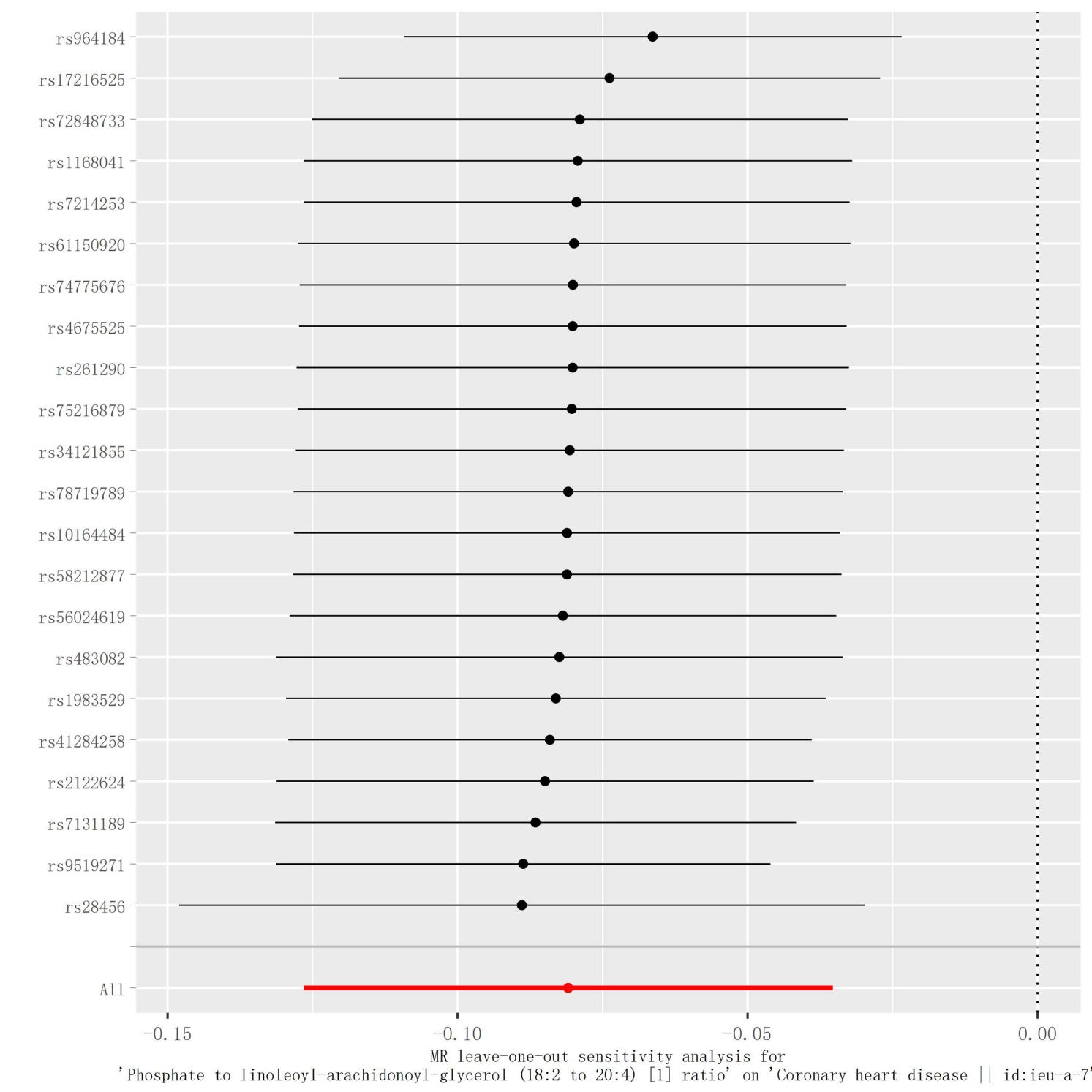

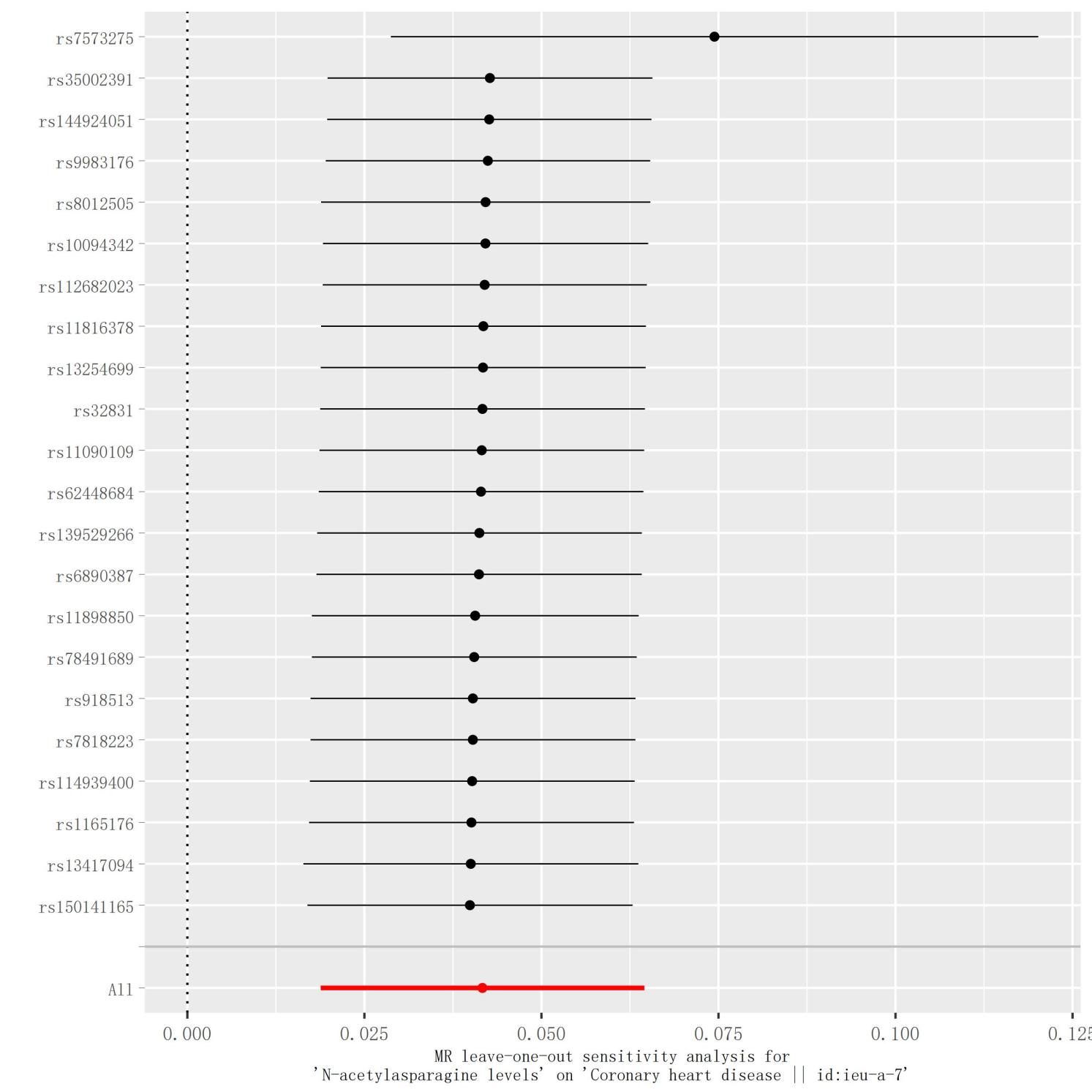

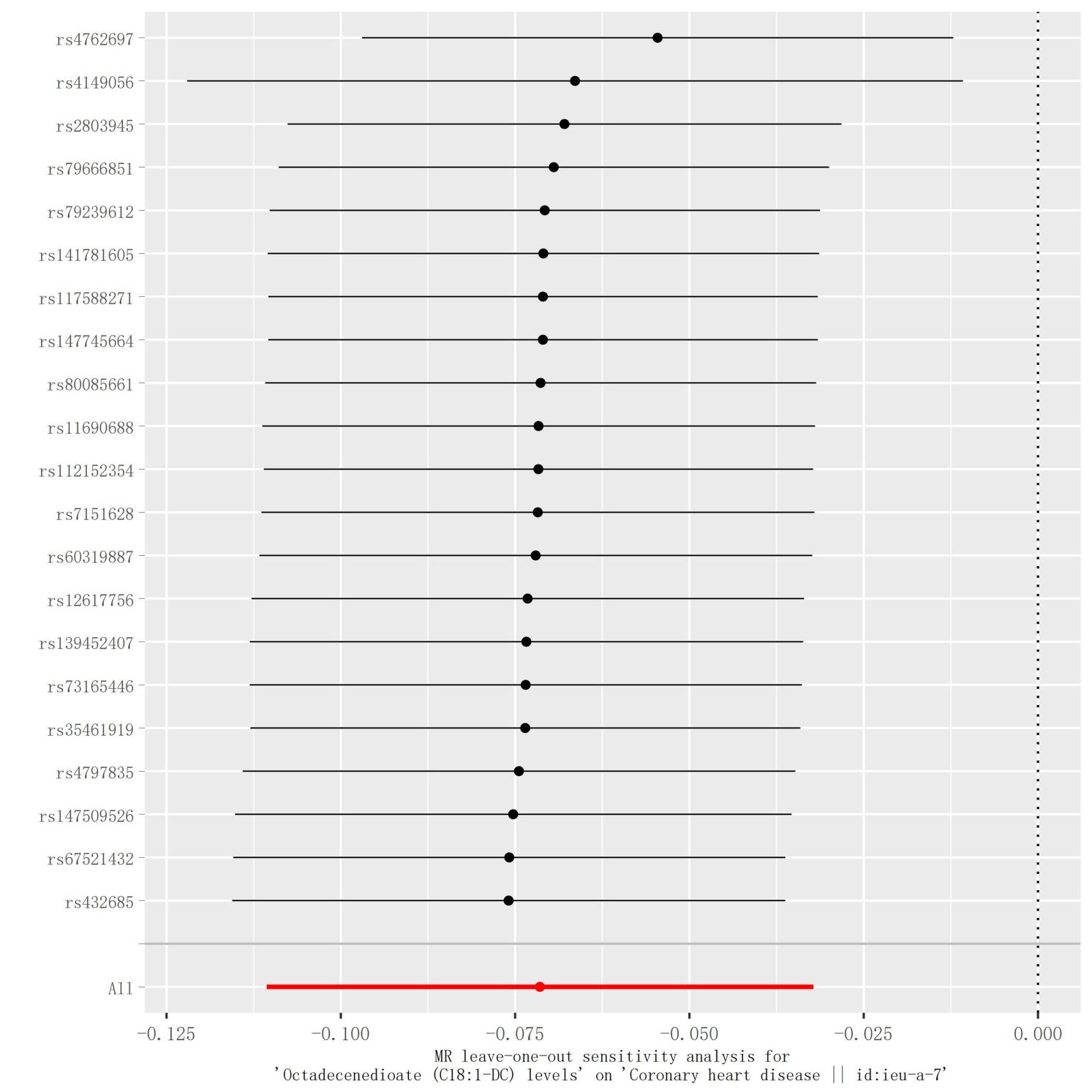

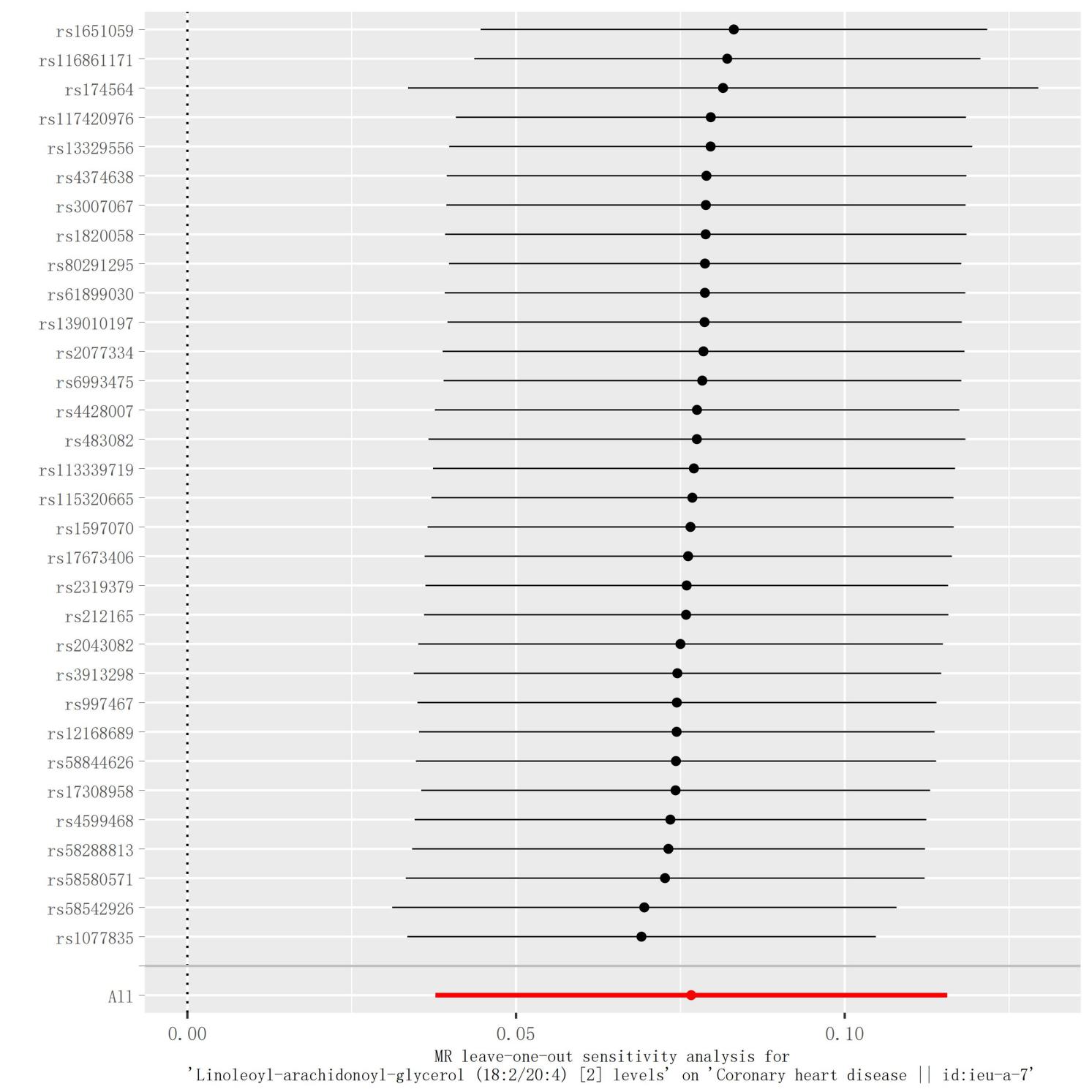

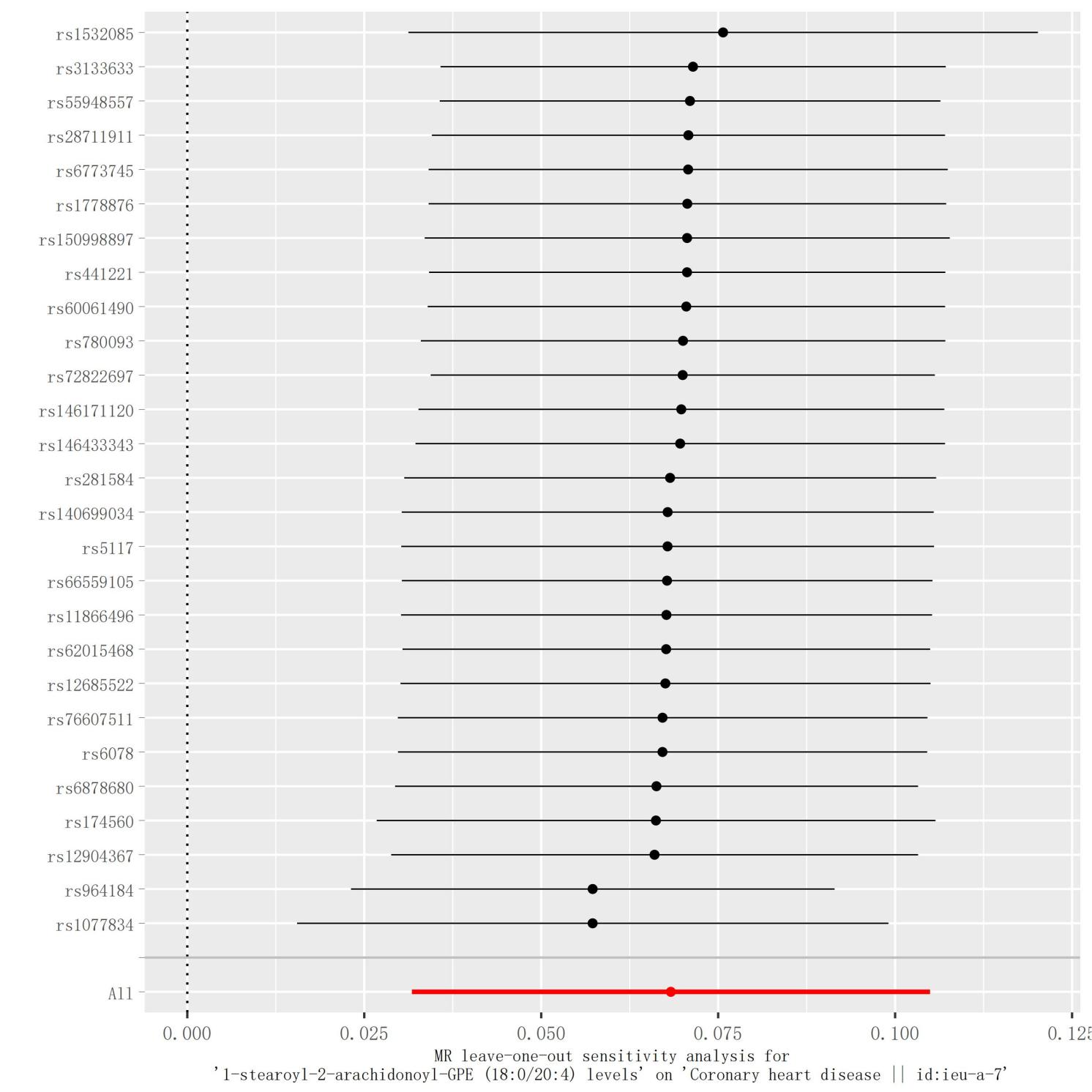

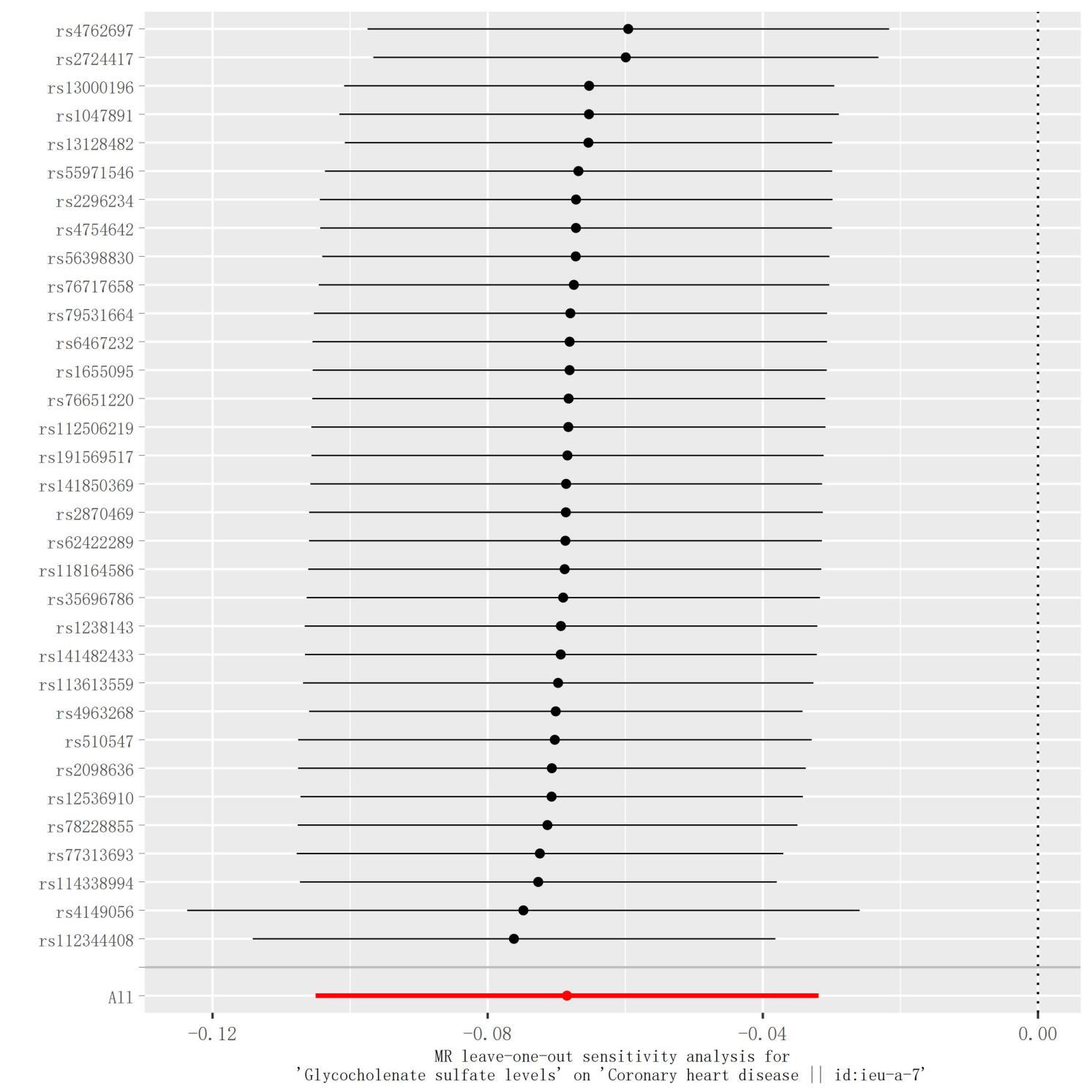


**Supplementary Figures S1–S8.** Results of leave-one-out analyses for specific metabolites and CHD. The red line indicates the MR results of the IVW analysis, with no significant difference in causal estimates between blood metabolites and CHD after removal of either IV.


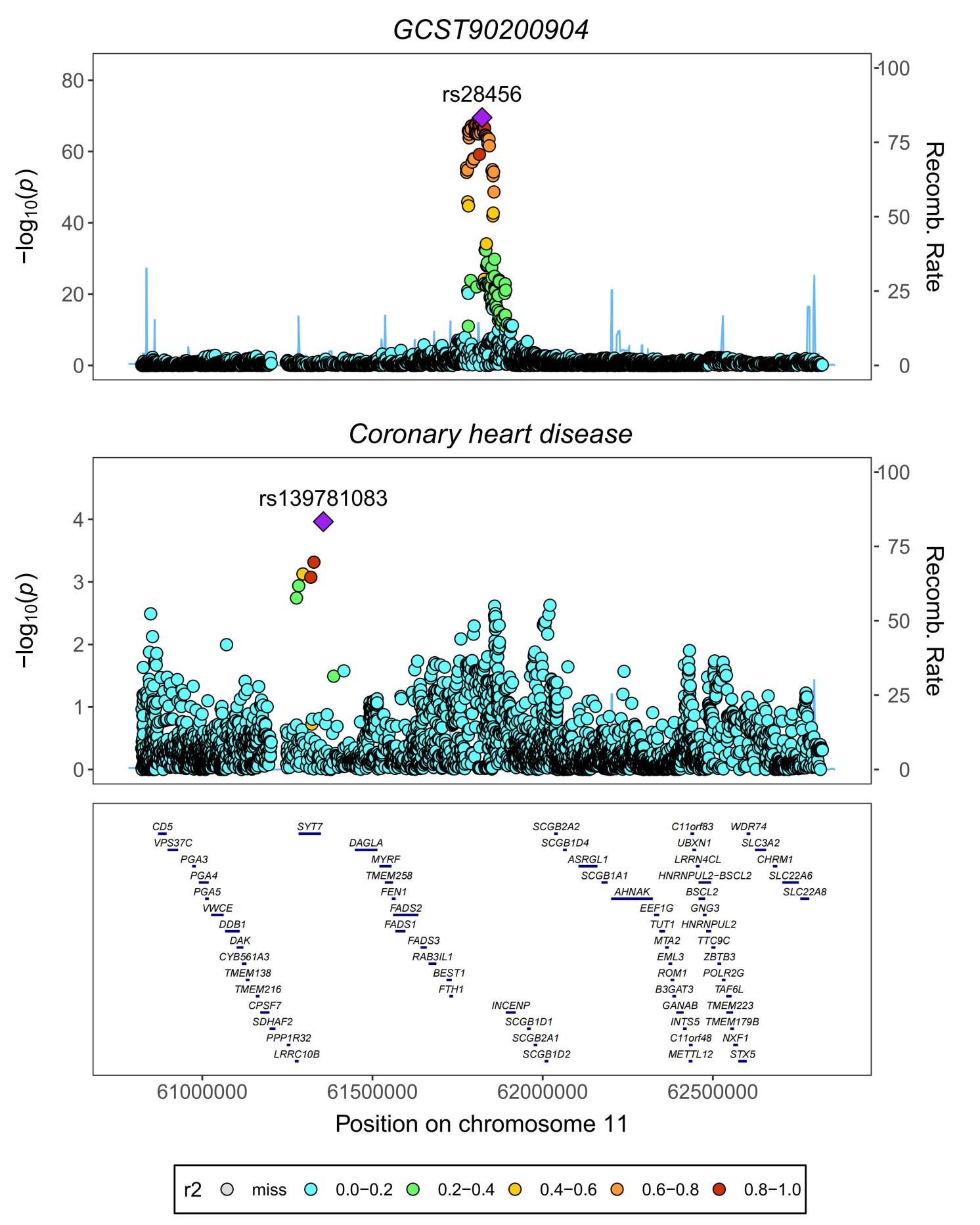

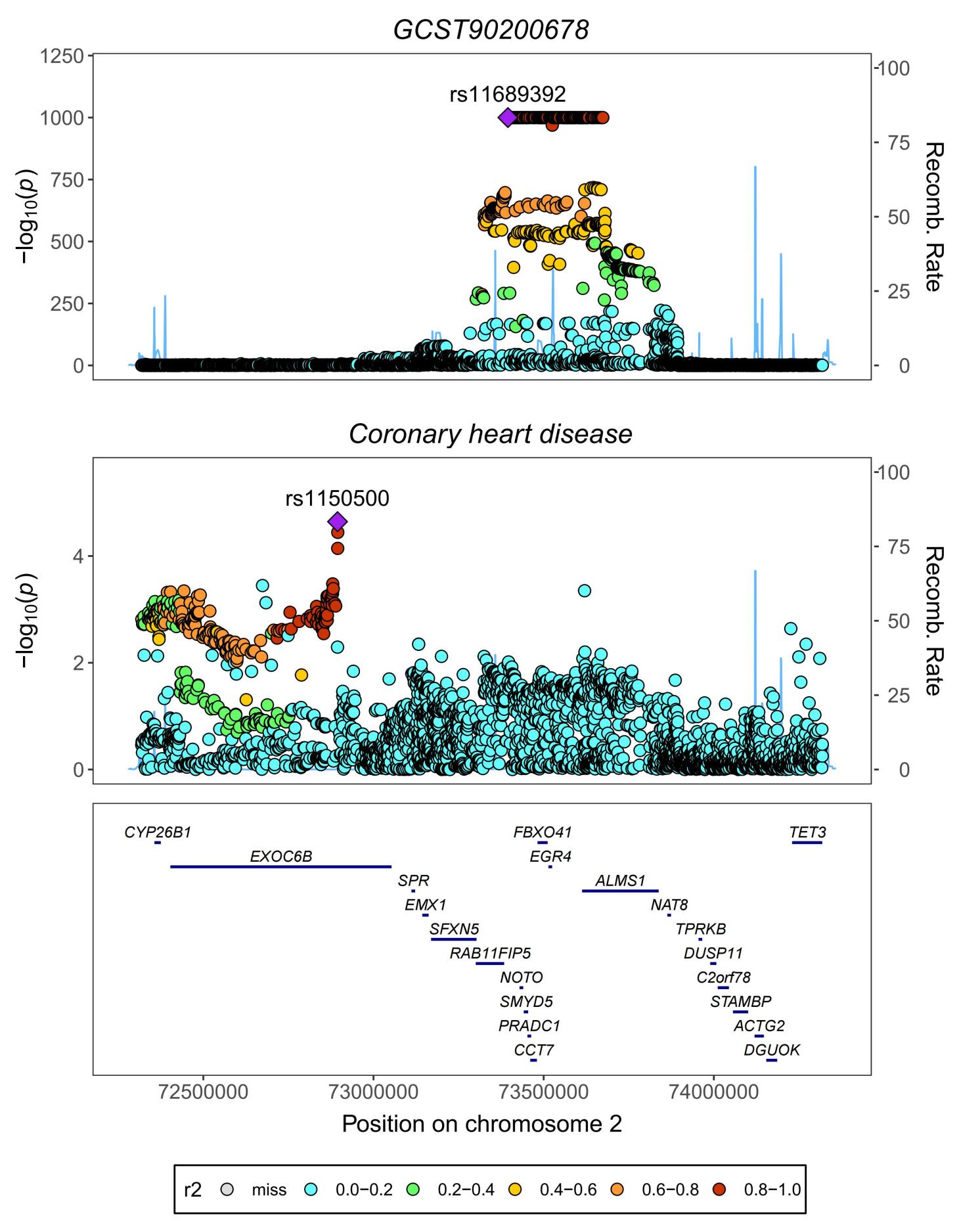

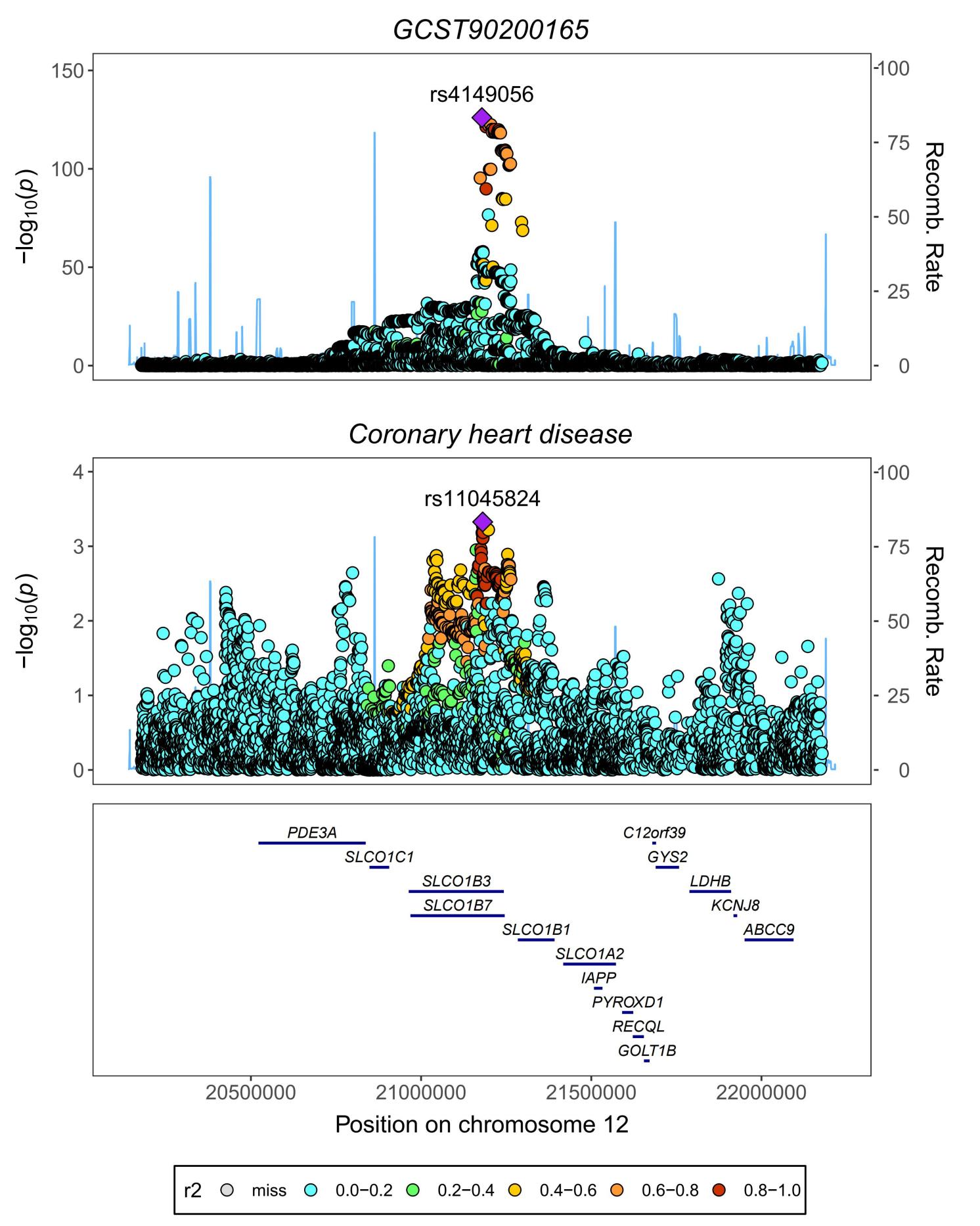

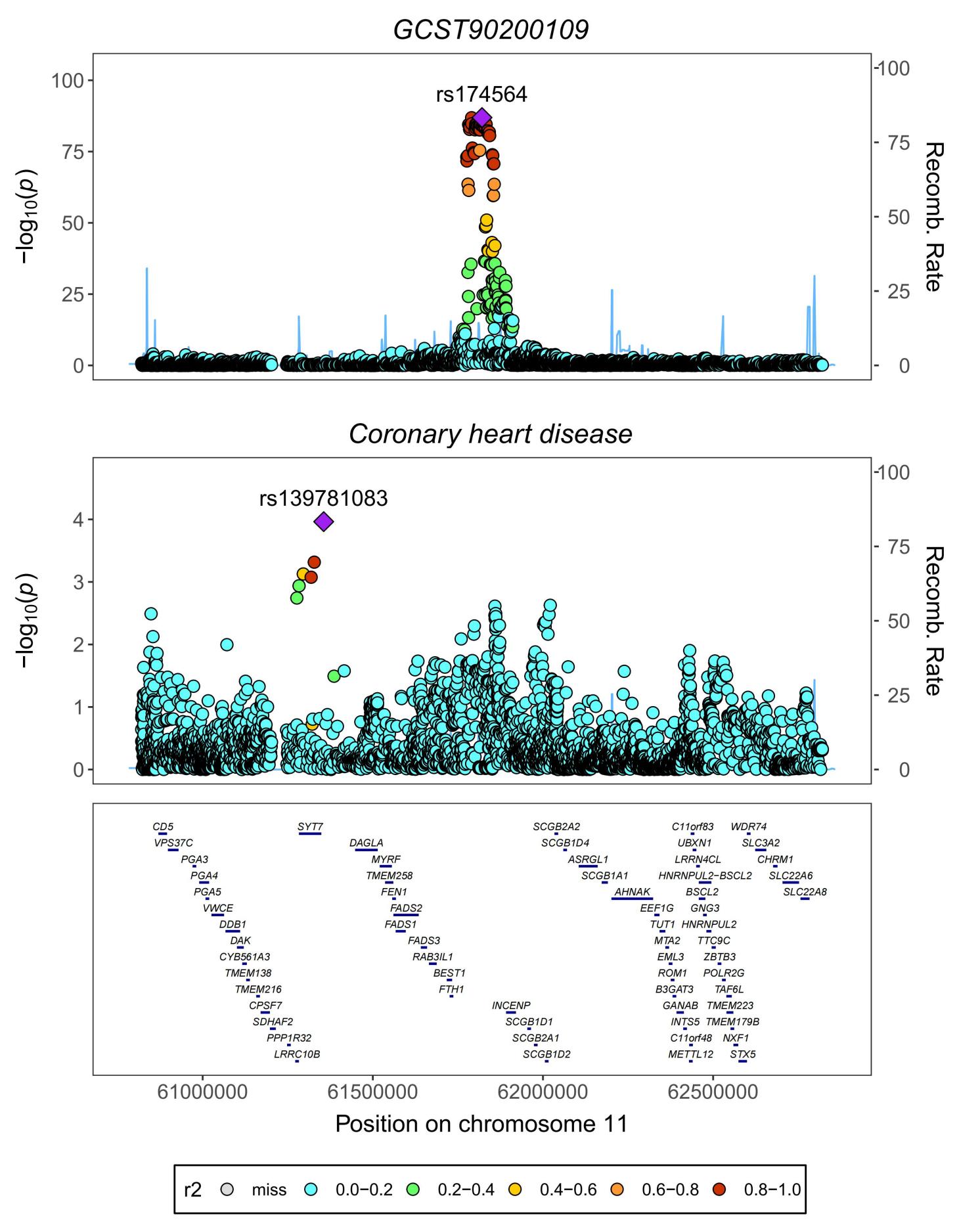

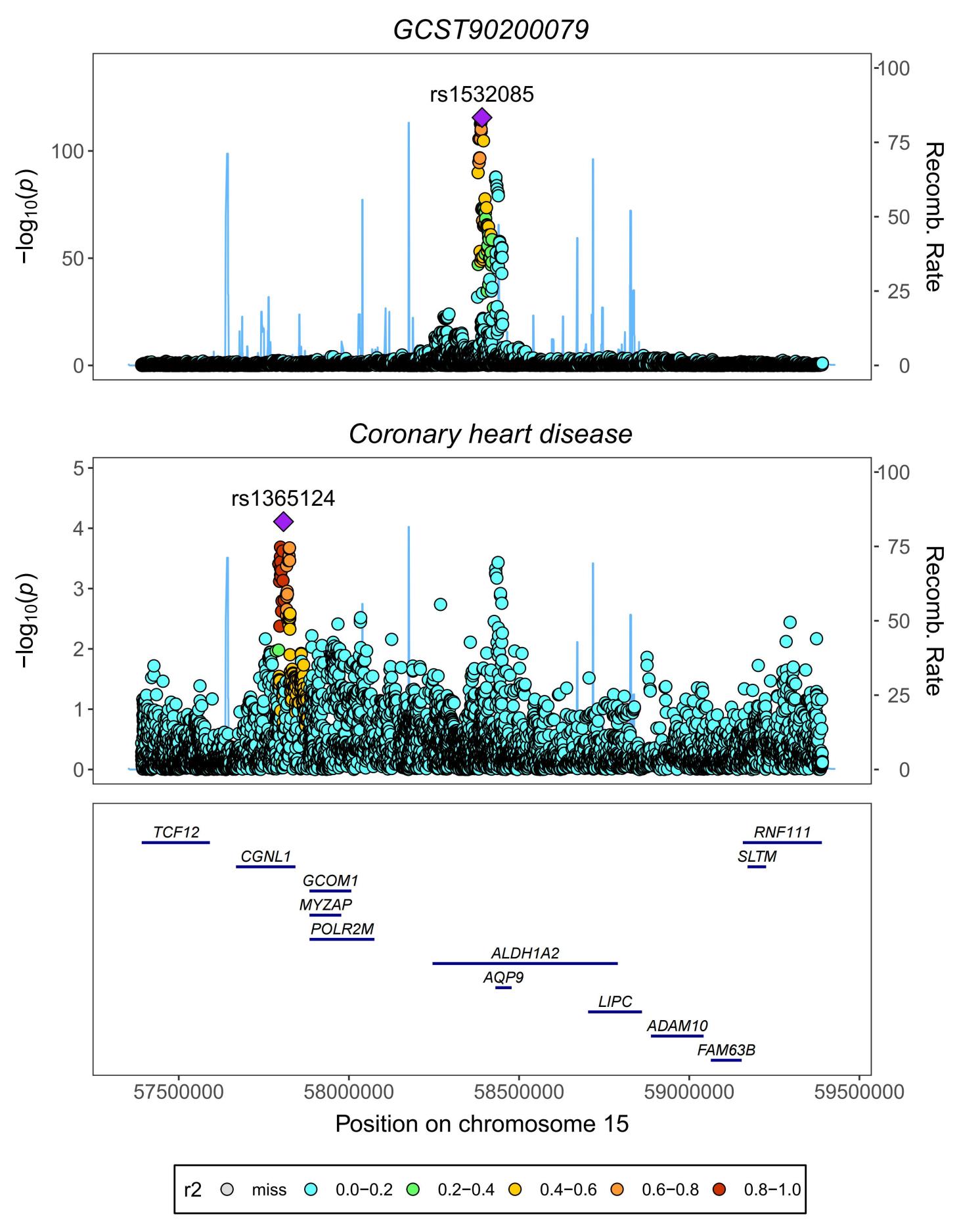

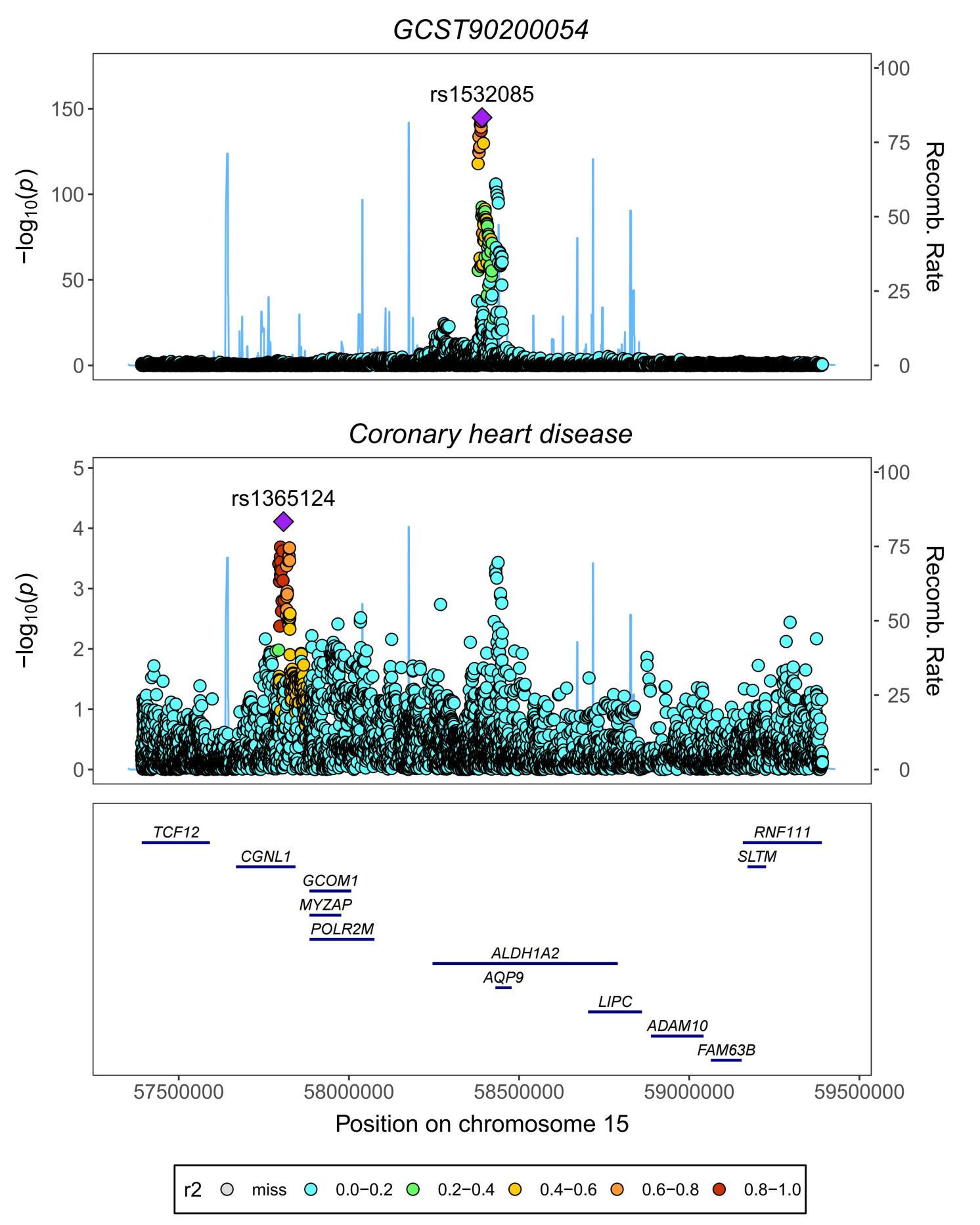

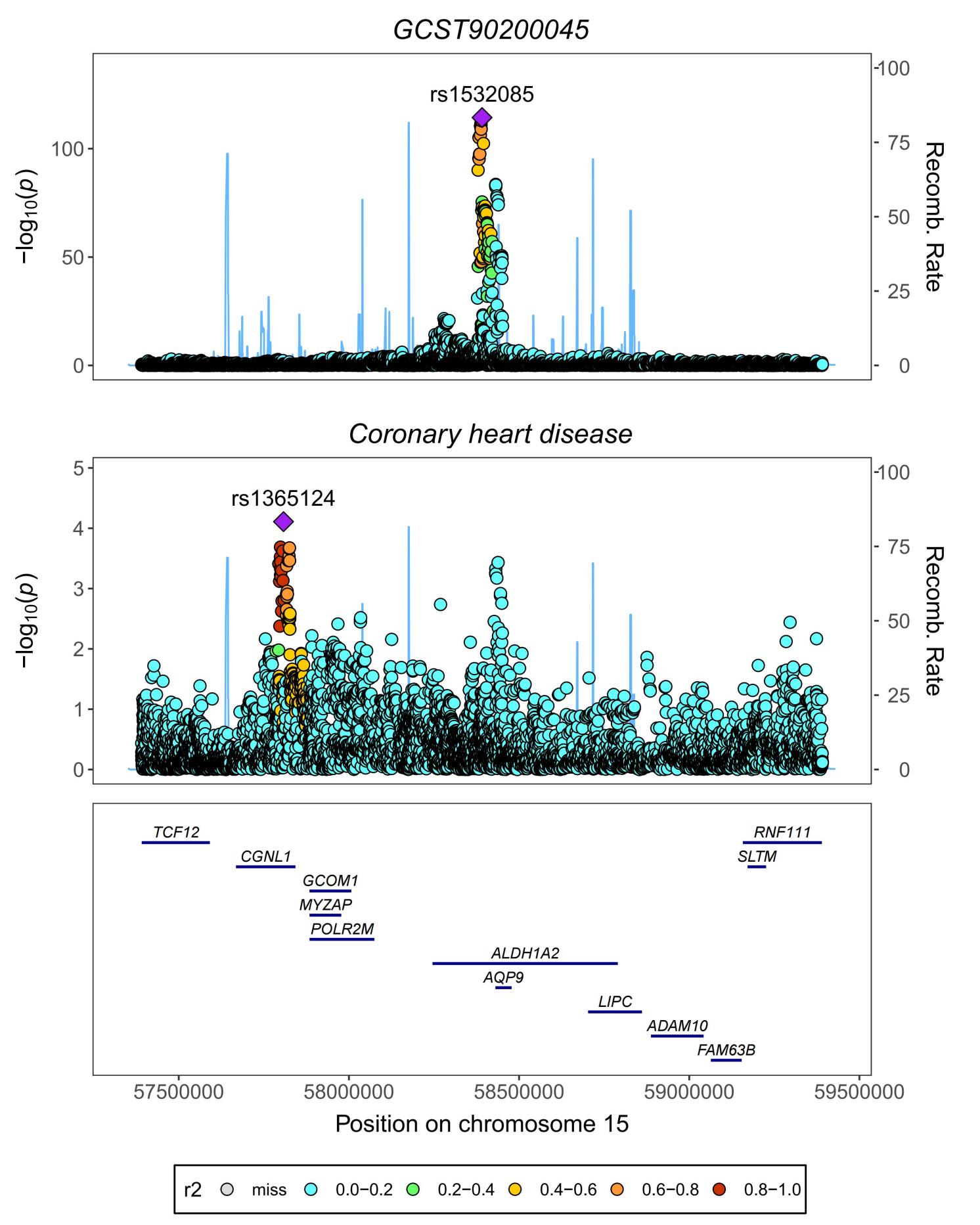

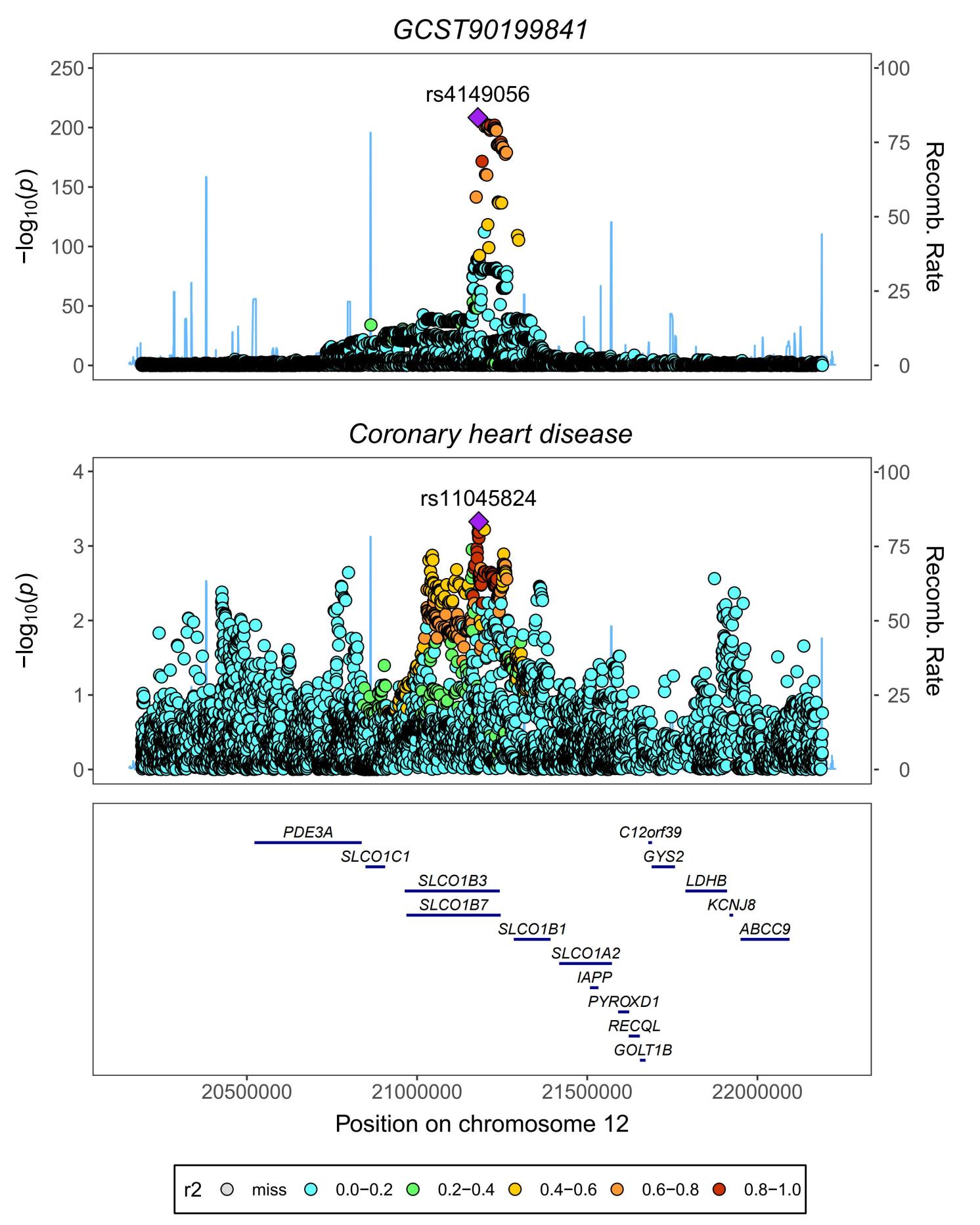


**Supplementary Figures S9–S16**. Regional association plots for the colocalization analysis of metabolites with Coronary Heart Disease (CHD). The lead single nucleotide polymorphism (SNP) is marked as a purple diamond. SNPs within ± 1000 kb of the protein quantitative trait locus are included, with a prior probability (p12) of 1e-5, indicating the likelihood of a SNP being associated with metabolites and CHD.
